# Grayscale, subjective color Doppler, combined grayscale with subjective color Doppler in predicting thyroid carcinoma: a retrospective analysis

**DOI:** 10.1016/j.bjorl.2020.05.024

**Published:** 2020-07-09

**Authors:** Minxin Wang, Xiaoting Wang, Hongsheng Zhang

**Affiliations:** a Weihai Central Hospital, Department of Ultrasound, Shandong, China; b Weihai Central Hospital, Department of Imaging, Shandong, China

**Keywords:** Color Doppler ultrasound, Fine-needle aspiration cytology, Grayscale ultrasound, Suspicious malignant nodule, Thyroid carcinoma

## Abstract

**Introduction:**

Fine needle aspiration cytology is preferred for thyroid nodules preoperatively, but has disadvantages of false-negative and false-positive results.

**Objective:**

To compare the diagnostic performance of grayscale ultrasound, subjective color Doppler ultrasound, and combined features of grayscale ultrasound and subjective color Doppler ultrasound in predicting thyroid carcinoma, using results of the fine needle aspiration cytology as the reference standard.

**Methods:**

Data from gray-scale ultrasound images, subjective color Doppler ultrasound images, and the fine needle aspiration cytology of 325 nodules of 250 patients (age ≥ 18 years) were collected and analyzed. Hypo-echogenicity than adjacent strap muscle, micro-lobulated or irregular margins, micro- or mixed calcifications, and taller-than-wide shapes were considered as a suspicious malignant nodule in grayscale ultrasound. Marked vascularity was considered as a suspicious malignant nodule in color Doppler ultrasound. The Bethesda system for classification of thyroid nodules was used for cytopathology.

**Results:**

With respect to the results of fine-needle aspiration cytology for detecting suspicious malignant nodules, for grayscale ultrasound, subjective color Doppler ultrasound, and combined gray-scale with subjective color Doppler ultrasound, sensitivities were 0.564, 0.600 and 0.691, respectively and accuracies were 0.926, 0.919 and 0.959, respectively. Suspicious malignant nodules detectability for grayscale ultrasound, subjective color Doppler ultrasound, and combined gray-scale with subjective color Doppler ultrasound were 0.09–0.56 diagnostic confidence, 0.08–0.61 diagnostic confidence, and 0.063–0.7 diagnostic confidence, respectively.

**Conclusions:**

The combined gray-scale with subjective color Doppler ultrasound-guided fine-needle aspiration biopsies are recommended for the diagnosis of thyroid carcinoma.

Level of Evidence: III.

## Introduction

Thyroid carcinoma is a very common malignancy in clinical practice.^1^ Imaging modalities make it possible to increase the detection of thyroid nodules.^2^ Diagnostic modalities can effectively predict the presence or absence of nodule(s) in neck imaging in two out of three patients, and can help in clinical evaluation of thyroid nodules.^3^ Fine needle aspiration cytology is preferred for thyroid nodules preoperatively^1^ but has disadvantages of possible false-negative and false-positive results. Florid atypia is responsible for false-negative and positive results in fine-needle aspiration cytology,^4^ while real-time ultrasound can predict size, numbers, and site(s) of thyroid nodules.^5^

The 2015 American thyroid association management guidelines recommended gray-scale ultrasound for thyroid nodules^6^ but its features of malignant and benign nodules are overlapped.^7^ While color Doppler ultrasound allows visualization of minute vessels with slow blood velocity to predict characteristics of the tumor^8^ and improves the sensitivity of grayscale ultrasound^1^ but a prospective study reported that the performance of grayscale ultrasound combined with color Doppler does not improve diagnostic performance of the gray-scale ultrasound.^9^ There exists no clear consensus available for benign and suspicious nodules in color Doppler ultrasound examinations.^10^

The objective of the study was to compare the diagnostic performance of the grayscale ultrasound, subjective color Doppler ultrasound, and combined features of grayscale ultrasound and subjective color Doppler ultrasound in predicting thyroid carcinoma, using results of fine-needle aspiration cytology as the reference standard.

## Methods

### Ethics approval and consent to participate

The designed protocol (WCH/CL/31/19 dated 22 October 2019) was approved by the institutional review board and the medical council of the country. The study reporting adheres to the law of the country. An informed consent form was signed by all patients regarding diagnosis and the publication of the study, including personal data and images irrespective of time and language during hospitalization. Approval was obtained from competing authorities before the collection of data.

### Study population

**Figure 1:**
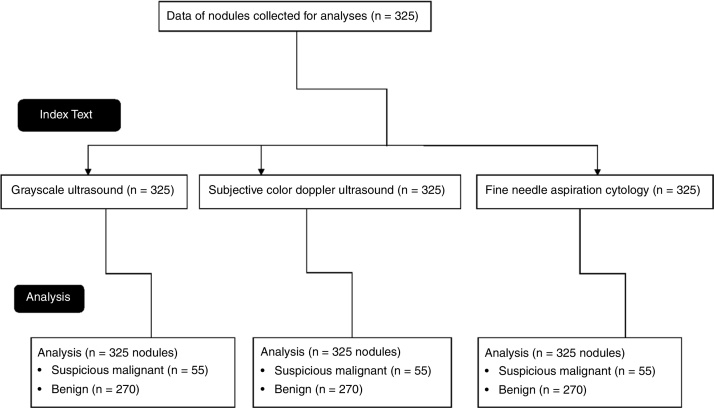
Flow diagram of the study.

Gray-scale ultrasound and color Doppler ultrasound images of 325 nodules of 250 patients (age ≥ 18 years) admitted from 15 January 2018 to 10 September 2019 at the parent hospital and the referring hospitals were collected after institutional approval. All patients had been subjected to fine-needle aspiration cytology during their diagnosis and treatment of thyroid carcinoma (Fig. 1). Female patient numbers exceeded that of male patients. The other demographical and clinical conditions of the enrolled patients are presented in Table 1.

**Table 1:** The demographical and clinical conditions of the enrolled patients.

Characteristics		Value
Nodules subjected to analysis		325
Patients included in the analysis		250
Age (years)	Minimum	19
	Maximum	71
	Mean ± SD	48.55 ± 7.54
Gender	Male	53 (21)
	Female	197 (79)
Ethnicity	Han Chinese	227 (91)
	Mongolian	20 (8)
	Tibetan	3 (1)
Nodules per patient mean (range)		1.3 (1–4)
Family history of thyroid carcinoma		12 (5)

Categorical data demonstrate as frequency (percentage) and continuous data demonstrate as mean ± SD.

### Ultrasound examinations

All patients were instructed to lay down on the bed in a supine position using Versana Premier (GE Healthcare system, Chicago, IL, USA) and EPIQ Elite (Philips Medical System, Chicago, IL, USA) equipment with 12 MHz linear probe, gray-scale and color Doppler images were derived in the single setting by the radiologists (a minimum of 3 years of experience of thyroid images) of the institutes. Standard transverse and longitudinal ultrasound images were evaluated.

### Image analysis

**Figure 2:**
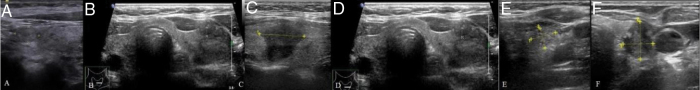
Grayscale ultrasound diagnoses of the thyroid nodules. A, Hypoechogenicity than adjacent strap muscle, B, Micro-lobulated, C, Irregular margins, D, Microcalcifications, E, Mixed-calcifications, F, The taller-than-wide shape in greater anteroposterior dimension.

In the grayscale ultrasound, marked hypoechogenicity than adjacent strap muscle (Fig. 2A), micro-lobulated (Fig. 2B) or irregular margins (Fig. 2C), micro (Fig. 2D) or mixed calcifications (Fig. 2E), and taller-than-wide shapes (anteroposterior diameter longer than the transverse diameter on a transverse/longitudinal plane) (Fig. 2F) were considered as a suspicious malignant nodule. For thyroid nodules, the absence of the above features was considered as benign.^11^

**Figure 3:**
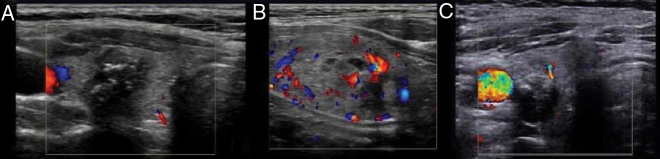
Subjective color Doppler ultrasound diagnosis. A, Avascularity; B, Vascularity ≤ 25% of the nodule circumference; C, Flow pattern is greater than that of the surrounding part.

In color Doppler images, avascularity (Fig. 3A) or peri-nodular flow (Fig. 3B) (vascularity ≤ 25% of the nodule circumference) was considered as a benign nodule and marked vascularity (flow pattern was greater than that of surrounding part) (Fig. 3C) was considered as suspicious for malignant nodules.^1^

Ultrasound technologists (a minimum of 5 years of experience of thyroid imaging) of the institutes were involved in the image analyses.

### Fine needle aspiration biopsies

In a supine position, the skin of the neck was sterilized with antiseptics. A 25G needle was used to puncture the skin and material from the thyroid was collected a in 10 mL aspirator. The collected sample was sent to the pathological laboratory for analysis. Biopsy evaluation was performed by pathologists (a minimum of 3 years of experience) of the institutes.

### Pathology

**Figure 4:**
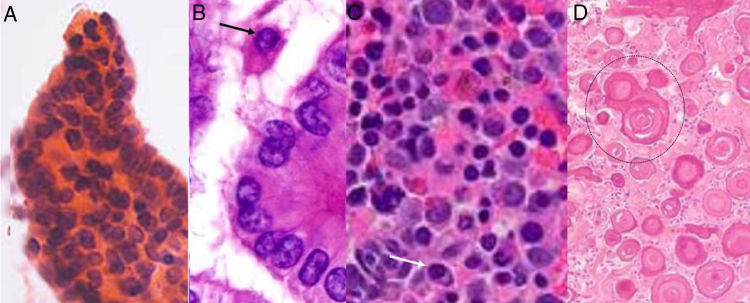
Histopathological features of suspicious malignant nodules. A, True papillae; B, Nuclear pseudo inclusions (black arrow); C, Mild nuclear irregularity (white arrow); and D, Psammoma bodies (black circle) (Hematoxylin & Eosin staining).

Biopsy samples were analyzed by cytopathologists (a minimum of 3 years of experience) of the institutes. The grading of lesions was performed as per the 2017 Bethesda System for the classification of thyroid nodules.^12^ True papillae (Fig. 4A), nuclear pseudo inclusions (Fig. 4B), mild nuclear irregularity (Fig. 4C), and psammoma bodies (Fig. 4D) in histopathological features were considered as suspicious malignant nodules. If these features were absent, they were considered benign nodules.^13^

### Diagnostic parameters

The ratio of true positive suspicious malignant nodules detected through imaging modality to those detected through fine needle aspiration cytology considered as sensitivity. The ratio of true positive benign nodules detected through imaging modality to those detected through fine needle aspiration cytology was considered as accuracy.

### Beneficial score analysis

Beneficial score analysis for grayscale ultrasound, subjective color Doppler ultrasound, and combined gray-scale with subjective color Doppler for decision-making in fine needle aspiration biopsies was calculated as per Eqs. (1) and (2).^14^

(1) $$\text{B}\text{e}\text{n}\text{e}\text{f}\text{i}\text{c}\text{i}\text{a}\text{l}\;\text{s}\text{c}\text{o}\text{r}\text{e}\;\text{a}\text{n}\text{a}\text{l}\text{y}\text{s}\text{i}\text{s}=\frac{\text{T}\text{r}\text{u}\text{e}\;\text{p}\text{o}\text{s}\text{i}\text{t}\text{i}\text{v}\text{e}\;\text{s}\text{u}\text{s}\text{p}\text{i}\text{c}\text{i}\text{o}\text{u}\text{s}\;\text{m}\text{a}\text{l}\text{i}\text{g}\text{n}\text{a}\text{n}\text{t}\;\text{n}\text{o}\text{d}\text{u}\text{l}\text{e}\;\text{d}\text{e}\text{t}\text{e}\text{c}\text{t}\text{e}\text{d}}{\text{T}\text{o}\text{t}\text{a}\text{l}\;\text{n}\text{u}\text{m}\text{b}\text{e}\text{r}\text{s}\;\text{o}\text{f}\;\text{n}\text{o}\text{d}\text{u}\text{l}\text{e}\text{s}\;\text{a}\text{n}\text{a}\text{l}\text{y}\text{z}\text{e}\text{d}}-(\frac{\text{F}\text{a}\text{l}\text{s}\text{e}-\text{p}\text{o}\text{s}\text{i}\text{t}\text{i}\text{v}\text{e}\;\text{s}\text{u}\text{s}\text{p}\text{i}\text{c}\text{i}\text{o}\text{u}\text{s}\;\text{m}\text{a}\text{l}\text{i}\text{g}\text{n}\text{a}\text{n}\text{t}\;\text{n}\text{o}\text{d}\text{u}\text{l}\text{e}\text{s}\;\text{d}\text{e}\text{t}\text{e}\text{c}\text{t}\text{e}\text{d}}{\text{T}\text{o}\text{t}\text{a}\text{l}\;\text{n}\text{u}\text{m}\text{b}\text{e}\text{r}\text{s}\;\text{o}\text{f}\;\text{n}\text{o}\text{d}\text{u}\text{l}\text{e}\text{s}\;\text{a}\text{n}\text{a}\text{l}\text{y}\text{z}\text{e}\text{d}}\;\times \text{R}\text{i}\text{s}\text{k}\;\text{o}\text{f}\;\text{u}\text{n}\text{d}\text{e}\text{r}\text{d}\text{i}\text{a}\text{g}\text{n}\text{o}\text{s}\text{i}\text{s}\;$$

(2) $$\text{R}\text{i}\text{s}\text{k}\;\text{o}\text{f}\;\text{u}\text{n}\text{d}\text{e}\text{r}\text{d}\text{i}\text{a}\text{g}\text{n}\text{o}\text{s}\text{i}\text{s}=\frac{\text{D}\text{i}\text{a}\text{g}\text{n}\text{o}\text{s}\text{i}\text{s}\;\text{c}\text{o}\text{n}\text{f}\text{i}\text{d}\text{e}\text{n}\text{c}\text{e}\;\text{a}\text{b}\text{o}\text{v}\text{e}\;\text{w}\text{h}\text{i}\text{c}\text{h}\;\text{f}\text{i}\text{n}\text{e}-\text{n}\text{e}\text{e}\text{d}\text{l}\text{e}\;\text{a}\text{s}\text{p}\text{i}\text{r}\text{a}\text{t}\text{i}\text{o}\text{n}\;\text{b}\text{i}\text{o}\text{p}\text{s}\text{y}\;\text{w}\text{a}\text{s}\;\text{p}\text{e}\text{r}\text{f}\text{o}\text{r}\text{m}\text{e}\text{d}}{1-\text{D}\text{i}\text{a}\text{g}\text{n}\text{o}\text{s}\text{i}\text{s}\;\text{c}\text{o}\text{n}\text{f}\text{i}\text{d}\text{e}\text{n}\text{c}\text{e}\;\text{a}\text{b}\text{o}\text{v}\text{e}\;\text{w}\text{h}\text{i}\text{c}\text{h}\;\text{f}\text{i}\text{n}\text{e}-\text{n}\text{e}\text{e}\text{d}\text{l}\text{e}\;\text{a}\text{s}\text{p}\text{i}\text{r}\text{a}\text{t}\text{i}\text{o}\text{n}\;\text{b}\text{i}\text{o}\text{p}\text{s}\text{y}\;\text{w}\text{a}\text{s}\;\text{p}\text{e}\text{r}\text{f}\text{o}\text{r}\text{m}\text{e}\text{d}}\;$$

True positive suspicious malignant nodule: suspicious malignant nodule detected by imaging modality and detected by fine-needle aspiration cytopathology.

False-positive suspicious malignant nodule: suspicious malignant nodule detected by imaging modality but not detected by fine-needle aspiration cytopathology.

### Statistical analyses

InStat version Window 3.01, GraphPad, San Diego, CA, USA was used for statistical analyses purpose. For categorical variables, the Chi-square Independence test was performed and for a continuous variable^15^ and an independent two-sample *t*-test was performed.^9^ Univariate following multivariate analysis was performed for association of false predictive values and histopathological features. Results were considered significant at a 95% Confidence Level.

## Results

**Figure 5:**
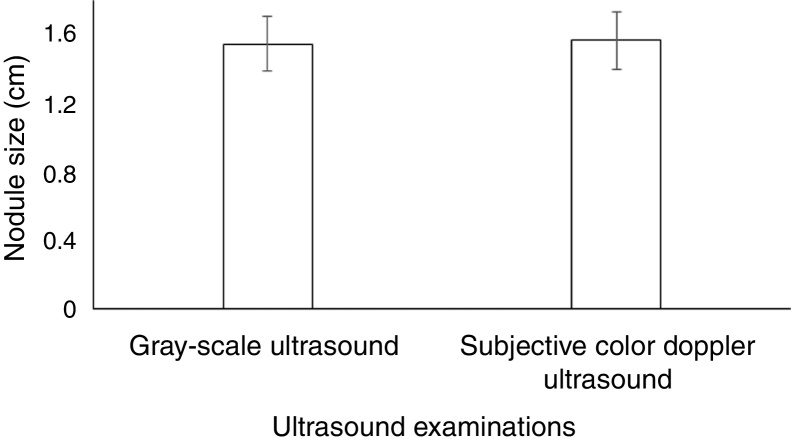
Nodule size distributions by ultrasound examinations. Data demonstrate mean ± SD. Data of 325 nodules were used for analysis. An Independent two-sample *t*-test was performed for statistical analysis. A *p* < 0.05 was considered significant.

There was no significant difference between nodule size detected by gray-scale ultrasound and subjective color Doppler ultrasound (1.54 ± 0.16 cm vs. 1.56 ± 0.17 cm, *p* = 0.872) (Fig. 5).

### Diagnostic parameters

With respect to the results of fine-needle aspiration cytology for detecting suspicious malignant nodules, grayscale ultrasound, subjective color Doppler ultrasound, and combined gray-scale with subjective color Doppler, sensitivities were 0.564, 0.600, and 0.691, respectively and accuracies were 0.926, 0.919, and 0.959, respectively. There was no significant difference between the combined gray-scale with subjective color Doppler ultrasound and fine-needle aspiration cytopathology for true positive suspicious malignant nodules (*p* = 0.073) and true positive benign nodules (*p* = 0.314) but it reported significant numbers of false-positive suspicious malignant nodules (*p* < 0.0001) and false-positive benign nodules (*p* = 0.003). The detailed diagnostic parameters are presented in Table 2.

**Table 2:** Diagnostic performance of ultrasound techniques.

Parameters	Fine-needle aspiration cytology	Grayscale ultrasound		Subjective color Doppler ultrasound		Combined grayscale with subjective color Doppler	
Total numbers of nodules analyzed	325	325	a*p*-Value	325	a*p*-Value	325	a*p*-Value
True positive suspicious malignant nodules detected	55 (17)	31 (10)	0.008	33 (10)	0.016	38 (12)^b^	0.073
True positive benign nodules detected	270 (83)	250 (77)^b^	0.062	248 (76)	0.041	259 (80)^b^	0.314
False positive suspicious malignant nodules detected	0 (0)	24 (7)	<0.0001	22 (7)	<0.0001	17 (5)	<0.0001
False positive benign nodules detected	0 (0)	20 (6)	<0.0001	22 (7)	<0.0001	11 (3)	0.003
Sensitivity	1	0.564	0.008	0.600	0.016	0.691^b^	0.073
Accuracy	1	0.926^b^	0.062	0.919^b^	0.067	0.959^b^	0.314

Data are shown as frequency (percentage).

A Chi-Square independence test was performed for statistical analysis.

A *p* < 0.05 was considered significant.

a With respect to fine-needle aspiration cytology.

b Insignificant difference with respect to fine-needle aspiration cytology.

### Beneficial score analysis

**Figure 6:**
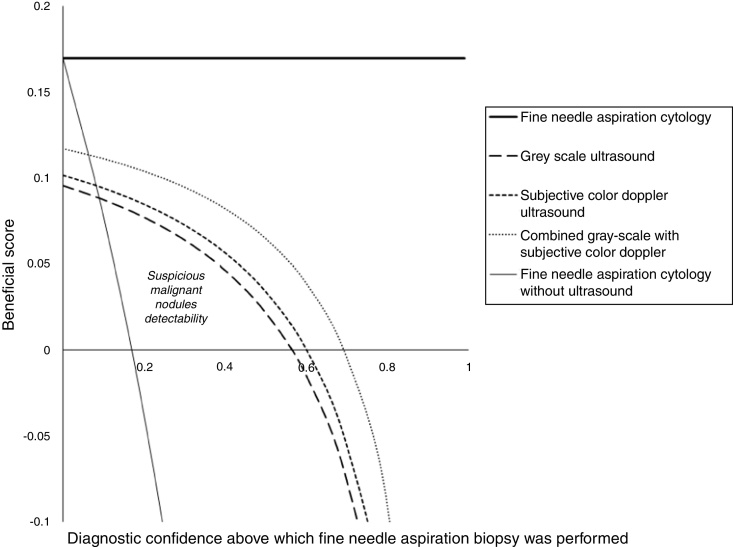
Beneficial score analysis. Ultrasound technologists (a minimum of 5 years of experience of thyroid images) of the institutes were involved in the image analyses.

Suspicious malignant nodule detectability for grayscale ultrasound, subjective color Doppler ultrasound, and subjective combined gray-scale with color Doppler ultrasound was 0.09–0.56 diagnostic confidence, 0.08–0.61 diagnostic confidence, and 0.063–0.7 diagnostic confidence, respectively (Fig. 6). Above 0.56, 0.61 and 0.7 diagnostic confidence grayscale ultrasound, subjective color Doppler ultrasound, and subjective combined gray-scale with color Doppler ultrasound had no diagnostic potential respectively and below 0.09, 0.08 and 0.063 diagnostic potential grayscale ultrasound, subjective color Doppler ultrasound, and subjective combined gray-scale with color Doppler ultrasound had the risk of overdiagnosis respectively.

### Pathological parameters detected by ultrasound imaging

Univariate analysis reported that gray-scale and subjective color Doppler ultrasound decreased false predictive values of all types of histopathological features (*p* > 0.0001 for all). However, multivariate analysis reported that gray-scale ultrasound decreased false predictive values for mild nuclear irregularity (*p* = 0.051) and psammoma bodies (*p* = 0.053). Subjective color Doppler ultrasound decreased false predictive values for nuclear pseudo inclusions (*p* = 0.052) and mild nuclear irregularity (*p* = 0.052). The combined grayscale with subjective color Doppler decreased false predictive values for nuclear pseudo- inclusions (*p* = 0.052), mild nuclear irregularity (*p* = 0.053), and psammoma bodies (*p* = 0.055). However, ultrasound imaging failed in reduction of false predictive values for true papillae (*p* < 0.05 for all modalities). The detailed association of false prediction due to imaging modalities values and histopathological features reported in fine-needle aspiration cytopathology are reported in Table 3.

**Table 3:** Association of false predictive due to imaging modalities values and histopathological features.

Ultrasound technique	Grayscale ultrasound			Subjective color Doppler ultrasound			Combined grayscale with subjective color Doppler		
False negative predictive values	44			44			28		
Histopathological parameters	OR	95% CL	*p*-Value	Odd ratio	95% CL	p-Value	OR	95% CL	*p*-Value
True papillae	0.72	0.78–0.89	0.043^a^	0.73	0.77–0.81	0.039^a^	0.61	0.57–0.82	0.042^a^
Nuclear pseudo inclusions	0.61	0.77–0.91	0.042^a^	0.74	0.72–0.86	0.051	0.72	0.71–0.82	0.052
Mild nuclear irregularity	0.62	0.56–0.92	0.051	0.65	0.79–0.85	0.052	0.73	0.73–0.85	0.053
Psammoma bodies	0.69	0.62–0.78	0.053	0.76	0.76–0.91	0.048^a^	0.71	0.71–0.89	0.055

OR, odd ratio, 95% CL, 95% confidence limit.

Results of true positive a predictive value was considered reference standard. Multivariate regression analysis. A *p* < 0.05 was considered significant.

a Significant parameter of histopathology responsible for false predictive value.

## Discussion

Grayscale ultrasound, subjective color Doppler ultrasound, combined gray-scale with color subjective Doppler ultrasound had 0.564, 0.6 and 0.691 sensitivities. The addition of subjective color Doppler to grayscale moderately increases the sensitivity of diagnosis. The results of the study are consistent with prospective studies,^1^, ^16^, ^17^, ^18^ quantitative analysis of color Doppler ultrasound,^19^ and cross-sectional studies^2^, ^15^ but not consistent with prospective studies.^9^, ^10^, ^20^ The reason behind contradicted results is that there exists an absence of universal authoritative guidelines that indicate a specific vascularity pattern in ultrasound examinations.^9^ Also, visual assessment of nodular vascularity is subjective and has inter- and intra-observer variations.^17^ Angiogenesis and proliferation of vessels in suspicious malignant nodule increases vascularity in the color Doppler ultrasound.^19^ The current study recommended a combined gray-scale with subjective color Doppler ultrasound guidance for the performance of fine-needle aspiration biopsies.

The grayscale ultrasound had comparatively higher false negative and false positive suspicious malignant nodules. The results of the current study were parallel with prospective studies.^9^, ^21^ The follicular variant of papillary carcinoma resembles benign nodules in grayscale ultrasound and mixed- calcifications of benign lesions were considered as a suspicious malignant nodule.^1^ In subjective color Doppler, there are fewer chances of false-negative malignancy because the suspicious malignancy was detected on the basis of vascularity.

With respect to fine-needle aspiration biopsies, grayscale ultrasound, subjective color Doppler ultrasound, and the combined subjective gray-scale with color Doppler ultrasound reported significant false-positive suspicious malignant nodules (*p* < 0.0001 for all). The results of the study were consistent with retrospective analysis,^14^ prospective studies,^1^, ^9^ and cross-sectional study.^15^ Besides suspicious malignant nodules, thyroid calcifications are reported in benign nodules and conventional ultrasound is unable to differentiate calcified benign colloidal echogenicities from micro- or mixed-calcifications of suspicious malignant nodules.^18^ Therefore, ultrasound techniques reported false-positive suspicious malignant nodules.

Accuracies of combined gray-scale with color Doppler ultrasound were 0.959. The results of the study were parallel with prospective studies.^9^, ^22^ The distribution inside nodules and adjacent thyroid parenchyma are possible to study in better detail by color Doppler ultrasound. Therefore, the addition of color Doppler ultrasound increases the accuracy of gray-scale ultrasound.

The combined subjective gray-scale with color Doppler ultrasound imaging failed in reduction of false predictive values for true papillae type of suspicious malignant thyroid carcinoma. True papillae are a major type of thyroid carcinoma. This is small-sized and has no posterior acoustic shadowing. Conventional ultrasound has difficulties in detection of true papillae thyroid carcinoma.^18^ Shear-wave ultrasound elastography could be useful in such conditions.

In the limitations of the study, for example, ultrasound elastography was not performed. Elastography only improves visualization of tissue elasticity or stiffness properties but cannot improve the diagnosis.^23^ The results of the histopathology of the surgical specimen were not included in the analyses. Grading systems have the advantage of allowing different criteria to be applied to each grade in order to decide whether to perform fine-needle aspiration biopsies. For example, different size cut-offs can be applied to different grades but in the current study, the suspicious malignant detectability did not determine the size of the nodules. An experienced radiologist is required for image analysis in thyroid carcinoma^7^ but the study did not perform inter-or intra-observer agreements. In grayscale ultrasound echogenicity, lobulation, calcifications, and shape were used to rule out suspicious malignancy. The other features, like spongiform and cystic characteristics, were not used to rule out suspicious malignancy. This was the main reason for the reporting of significant numbers of false-positive benign nodules for grayscale ultrasound (*p* < 0.0001), subjective color Doppler ultrasound (*p* < 0.0001), and subjective combined gray-scale with color Doppler ultrasound (*p* = 0.003). Also, the study did not use a pulsatility index, mean systolic velocity, and resistive index for diagnosis of malignancy, but these are not useful parameters for detecting suspicious malignant nodules.^15^ If the combined grayscale with subjective color Doppler ultrasound may increase sensitivity and accuracy but decreases specificity but data regarding specificity did not evaluate.

## Conclusions

Grayscale ultrasound and subjective color Doppler ultrasound evaluations are both non-invasive and useful diagnostic techniques in the diagnosis of thyroid suspicious malignant nodules. Subjective color Doppler ultrasound may increase the sensitivity and accuracy of grayscale ultrasound in the detection of suspicious malignant thyroid nodules. The combined grayscale with subjective color Doppler ultrasound-guided fine-needle aspiration biopsies is recommended in the diagnosis of thyroid carcinoma.

## Compliance with ethics standard

The designed protocol (WCH/CL/31/19 dated 22 October 2019) was approved by the Weihai Central Hospital review board and the medical council of China.

## Availability of data and materials

The datasets used and analyzed during the current study available from the corresponding author on reasonable request.

## Conflicts of interest

The authors declare no conflicts of interest.
